# The impact of immune-related adverse events on the outcome of advanced gastric cancer patients with immune checkpoint inhibitor treatment

**DOI:** 10.3389/fimmu.2024.1503316

**Published:** 2024-12-24

**Authors:** Tianhang Zhang, Haitao Lv, Jiasong Li, Shasha Zhang, Jingjing Zhang, Siqi Wang, Yingnan Wang, Zhanjun Guo

**Affiliations:** ^1^ Department of Rheumatology and Immunology, The Fourth Hospital of Hebei Medical University, Shijiazhuang, China; ^2^ Department of Hepatobiliary Surgery, The Second Hospital of Hebei Medical University, Shijiazhuang, China; ^3^ Department of Gastroenterology and Hepatology, The Fourth Hospital of Hebei Medical University, Shijiazhuang, China

**Keywords:** gastric cancer, immune checkpoint inhibitors, immune-related adverse events, therapeutic efficacy, multi-organ irAEs

## Abstract

**Background:**

The occurrence of immune-related adverse events (irAEs) seemed to be associated with better outcomes in advanced gastric cancer (AGC) patients. However, research focusing on the impact of the single-organ irAE (uni-irAE) or multi-organ irAEs (multi-irAEs) on the AGC outcome is relatively limited. In this study, we investigated individually the impact of the different irAEs on AGC survival as well as the co-occurrence patterns of multi-irAEs.

**Methods:**

The uni-irAE, multi-irAEs, and non-irAE were identified based on National Comprehensive Cancer Network (NCCN) guidelines. ICI efficacy for the disease control rate (DCR) and the objective response rate (ORR) was assessed based on the Response Evaluation Criteria in Solid Tumors (RECIST) Version 1.1. The association for the irAEs with progression-free survival (PFS) or overall survival (OS) was analyzed using the Kaplan–Meier method and Cox regression model. We also performed pairwise correlation analysis to identify co-occurrence patterns of multi-organ irAEs.

**Results:**

A total of 288 patients including 175 non-irAE, 73 uni-irAE, and 40 multi-irAE patients were evaluated for their association with AGC outcome. The irAEs patients displayed higher DCR (78.8% vs. 67.4%, *p*=0.037) when compared with those of non-irAE patients, and both uni-irAE patients (82.2% vs. 67.4%, *p*=0.019) and multi-irAE patients (72.5% vs. 67.4%, *p*=0.534) showed higher DCR than that of non-irAE patients. The multivariate analyses revealed that multi-irAEs was an independent risk factor for PFS (hazard ratio [HR] of 0.63, 95% confidence interval [CI] 0.41~0.96, *p*=0.031) and OS (HR 0.47, 95% CI 0.29~0.76, *p*=0.002), whereas the survival association for uni-irAE was not obtained. The analysis of the co-occurrence patterns for multi-irAEs revealed that the thyroid, adrenal gland, heart, skin, and lung irAEs exhibited a high risk of co-occurrence of multi-irAEs. The multivariate Cox regression analysis for organ-specific irAEs revealed that patients experiencing thyroid, adrenal gland, and skin irAEs had favorable survival outcomes compared with those without these irAEs.

**Conclusion:**

Multi-irAEs and some organ-specific irAEs can be used as predictive indicators for ICI treatment efficacy in AGC patients. The thyroid, adrenal gland, heart, skin, and lung irAEs are often accompanied by multi-irAE occurrence.

## Introduction

According to GLOBOCAN 2022, gastric cancer (GC) accounted for the fifth incidence with 968,000 novel cases and the fifth mortality with approximately 659,000 deaths among all malignant tumors (1). China is a high-risk area for GC with a crude incidence rate of 254.1 per 100,000 and a crude mortality rate of 184.4 per 100,000 (1, 2). Due to the atypical early symptoms of GC, most patients are already in the advanced stage when diagnosed (3). There are several available options for advanced gastric cancer (AGC) treatment such as systemic chemotherapy, targeted therapies, and immunotherapy (4). The National Comprehensive Cancer Network (NCCN) guidelines recommend a systemic chemotherapy regimen based on platinum combined with fluoropyrimidine drugs (5). Even with conventional chemotherapy, the survival probability for AGC is still less than 1 year (6). Although molecular targeted therapies, such as targeting human epidermal growth factor receptor 2 (HER2), vascular endothelial growth factor receptor 2 (VEGFR-2), and Claudin18.2, have achieved treatment improvement in recent years, not all patients’ tumor cells have specific targets (7). Thus, chemotherapy and molecular targeted therapy have a limited impact on the overall prognosis of AGC. The advent of immunotherapy particularly immune checkpoint inhibitors (ICIs) has revolutionized the treatment landscape in recent years (8). The Food and Drug Administration (FDA) has approved a number of anti-PD-1 antibodies for tumor treatment (9). Based on various phase III clinical trials, such as ATTRACTION-2 phase III, CheckMate-649, ORIENT-16, RATIONALE-305, and KEYNOTE-859, anti-PD-1 antibodies have transitioned from a third-line option to a first-line treatment in AGC patients (4, 10–16). ICI treatment significantly gives an improved overall prognosis for AGC patients by enhancing the host’s endogenous immune response against the tumor (8).

The surveillance mechanisms of the host immune system play an important role in eliminating cancer, whereas tumors can express inhibitory signal molecules to suppress the immune function so as to achieve immune evasion via binding to their receptors on lymphocytes in the tumor microenvironment (TME) (17). These inhibitory signal molecules and their corresponding receptors are commonly referred to as immune checkpoints including programmed cell death-1 (PD-1), programmed cell death ligand-1 (PD-L1), and cytotoxic T lymphocyte-associated 4 (CTLA-4) (18). ICIs work by blocking immune checkpoints to restore the ability of the lymphocytes for tumor elimination (18).

However, the response to ICIs varies significantly among patients, leading to a hot research area for predicting individual responses (19–21). Previous studies have found that the appearance of immune-related adverse events (irAEs) may serve as a predictive indicator of good treatment outcomes (22–27).

IrAEs occur from weeks to months after initiation of ICIs, affecting various organs and tissues such as the skin, gastrointestinal system, lungs, liver, adrenal gland, and thyroid with a single organ involved or multiple organs involved (8). Some irAEs such as immune-related pneumonia, myocarditis, and enteritis can be fatal if they are not handled timely (28, 29). Based on the number of affected organs, irAEs can be categorized as “single-organ irAE (uni-irAE)” (affecting only one organ) and “multi-organ irAEs (multi-irAEs)” (affecting more than one organ) (23). Potential mechanisms for the development of irAEs include the common antigen between malignant cells and normal cells, the cross-immune response, off-target effects, B-cell activation and antibody-mediated damage, inflammatory cytokine-mediated damage, and the loss of peripheral tolerance in T cells caused by dysfunction of immune inhibitory cells (30–32). The occurrence of irAEs may signify the activation of the immune system, leading to continued attacks on tumor cells and causing sustained regression of the tumor. Previous studies have found that the emergence of irAEs was recognized as a key indicator of improved effectiveness of ICI treatment in various tumor types, including melanoma and small cell lung cancer (SCLC) (22, 23, 33, 34). We and other researchers also revealed that the irAEs were associated with better OS and PFS in AGC patients (25, 27, 35).

However, the impact of irAEs on the treatment efficiency remains uncertain in AGC patients, so it does for the co-occurrence patterns of multi-irAEs (34). Therefore, we evaluated the impact of the occurrence of irAEs including uni-irAE, multi-irAEs, and organ-specific irAE on both the therapeutic efficacy and prognosis of AGC patients with ICI treatment in the present study. The specific co-occurrence patterns of irAEs across multiple organs and their prognostic implications were also evaluated.

## Methods

### Patients

This retrospective study containing 288 AGC patients who received anti-PD-1 antibodies (sintilimab, tislelizumab, camrelizumab, pembrolizumab, nivolumab, toripalimab, or serplulimab) as monotherapy or combination therapy (combined with chemotherapy, targeted drugs, or both) in the Fourth Hospital of Hebei Medical University between June 2019 and December 2023.

The inclusion criteria were as follows (1): histopathology confirmed adenocarcinoma GC before treatment initiation; (2) advanced unresectable tumor according to the American Joint Committee on Cancer criteria 8^th^ (36); (3) patients who received PD-1 monotherapy or combination therapy; (4) no pertinent infections, nor any signs of acute or chronic inflammation, were observed before commencing treatment; (5) Eastern Cooperative Oncology Group (ECOG) score ≤2; (6) at least one measurable lesion according to the Response Evaluation Criteria in Solid Tumors (RECIST) Version 1.1 (37); (7) complete pathological and survival data were collected; (8) the patient’s age was over 28 years old. The exclusion criteria were as follows: (1) patients combined with other tumor history or blood system diseases; (2) pathological types other than adenocarcinoma; (3) ECOG score ≥3 or multiple organ failure; (4) patients combined with autoimmune diseases before treatment; (5) patients who were allergic to the ingredients of PD-1.

The clinical characteristics including age, gender, ECOG score, tumor node metastasis (TNM) stage, family history, treatment lines, tumor site, tumor differentiation, treatment regimen, number of metastases, HER2 status, microsatellite mismatch repair (MMR) status, and PD-L1 expression were collected for analysis. All procedures were conducted in line with the Helsinki Declaration of 1964 and its later amendments and have been reviewed and sanctioned by the Ethics Committee of the Fourth Hospital of Hebei Medical University. Due to the retrospective design of the study, the requirement for informed consent was waived.

### Evaluation and classification of irAEs

The classification and grading system of irAEs are based on the National Cancer Institute’s Common Terminology Criteria for Adverse Events (CTCAE), version 5.0 (CTCAE_v5_Quick_Reference_8.5x11.pdf (cancer.gov)). They were identified by physicians or pharmacists. The inclusion criteria were as follows: (1) the emergence of adverse events after initiation of immunotherapy; (2) pathological test results (if available); (3) diagnosed based on baseline disease status; (4) clinical improvement following irAE targeting therapies. The exclusion criteria were as follows: (1) the exclusion of other potential diagnoses with similar clinical manifestations; (2) determined by physicians that the adverse reaction is not caused by other medications (such as chemotherapy). IrAEs were classified based on the organ system affected (such as the skin, gastrointestinal tract, lungs, endocrine system, musculoskeletal system, kidneys, liver, nervous system, hematological system, and eyes). IrAEs were graded according to CTCAE and ranged from grade 1 to grade 5, which referred to mild, moderate, severe, life-threatening, or fatal events, depending on the severity of symptoms and the support of certain serum biomarkers. We defined irAEs affecting only a single organ as “uni-irAE” and those involving more than two organs as “multi-irAEs”. Consequently, the patients were categorized into three groups: those without any irAEs (non-irAE), those with uni-irAE, and those with multi-irAEs. Additionally, we used another classification method: each organ-specific irAE patients vs. other patients (including all the patients except the corresponding organ-specific irAE patients) to evaluate the ICI treatment efficacy for each organ-specific irAE.

### Treatment efficiency assessment and follow-up

Patients received anti-PD-1 antibodies (monotherapy or combination therapy [combined with chemotherapy, targeted drugs, or both]) every 21 days until disease progression, clinical deterioration, unacceptable toxicity, or patient refusal. Body computed tomography (CT) scans or magnetic resonance imaging (MRI) scans were taken every 2–3 cycles to evaluate objective tumor response according to the RECIST Version 1.1 (37). Complete response (CR) was characterized by the total disappearance of the target lesion following treatment, whereas partial response (PR) referred to a decrease of 30% or more in the total diameter of each target lesion. Progressive disease (PD) was indicated by a minimum 20% increase in the sum of the long diameters of all target lesions, accompanied by an increase of more than 5 mm or the development of new lesions. Stable disease (SD) was defined as no change in the size of target lesions. The objective response rate (ORR) represented the proportion of patients exhibiting either a CR or PR out of all patients who received treatment. The disease control rate (DCR) reflected the percentage of patients achieving a CR, PR, or SD.

All patients were monitored through re-hospitalization, outpatient clinic visits, and telephone follow-ups until death or loss of contact for any reason. PFS was defined as the period from treatment initiation to the date of disease progression, death, or study cutoff. OS was defined as the time from treatment initiation to death from any cause or study cutoff. The end point of follow-up was 1 June 2024 or the date of death.

### Statistical analysis

Statistical analyses were performed using SPSS version 27.0 software (IBM Corp., Armonk, NY, USA) and GraphPad Prism software (version 9.5, GraphPad Software, San Diego, CA, USA). Differences between the groups were compared using Pearson’s chi-squared test or Fisher’s exact test for categorical variables. Survival analysis for irAEs patients was evaluated using the Kaplan–Meier method with the log-rank test. Univariate and multivariate survival analyses were performed using the Cox proportional hazard model to evaluate the impact of the occurrence of irAEs. Univariate and multivariate logistic regression analyses were performed to evaluate risk factors for the occurrence of multi-irAEs. Univariate regression logistic analyses were performed to determine the association between each organ-specific irAE and multi-irAEs’ development. The time to the onset of irAEs between the two groups was assessed using the Mann–Whitney U test. Both univariate and multivariate Cox proportional hazard model analyses for outcomes (OS and PFS) were only performed for each organ-specific irAE with a case number of more than 10 patients. The differences were considered statistically significant when the *p*-value was less than 0.05.

## Results

### IrAEs mediate treatment efficacy of ICIs

A total of 288 AGC patients were involved with anti-PD-1 antibodies administering including sintilimab, tislelizumab, camrelizumab, pembrolizumab, nivolumab, toripalimab, and serplulimab (**Supplementary Table S1**). Among these patients, 5 patients received ICI monotherapy, 209 patients received a combination of ICI and chemotherapy, 30 patients received a combination of ICI and targeted therapy, and 44 patients received a triple regimen of ICI, chemotherapy and targeted therapy (**Supplementary Table S1**).

These patients were classified as 175 (60.76%) of non-irAE, 73 (25.35%) of uni-irAE, and 40 (13.89%) of multi-irAEs. The patients’ baseline characteristics are shown in **Table 1**; no significant difference was observed among the non-irAE group, uni-irAE group, and multi-irAEs group in terms of age, gender, ECOG score, TNM stage, family history, treatment lines, tumor site, tumor differentiation, treatment regimen, number of metastases, HER2 status, MMR status, and PD-L1 expression.

**Table 1:** The baseline clinical characteristics of non-irAE, uni-irAE, and multi-irAE groups in AGC patients.

Variable	Non-irAE group No.(%)	Uni-irAE group No.(%)	Multi-irAEs group No.(%)	*p*-value
**Gender**				0.107
Female	38 (21.71)	25 (34.25)	9 (22.50)	
Male	137 (78.29)	48 (65.75)	31 (77.50)	
**Age**				0.613
18-44	15 (8.57)	3 (4.11)	2 (5.00)	
45-65	79 (45.14)	39 (53.42)	19 (47.50)	
>65	81 (46.29)	31 (42.47)	19 (47.50)	
**ECOG**				0.889
≤1	105 (60.00)	46 (63.01)	25 (62.50)	
>1	70 (40.00)	27 (36.99)	15 (37.50)	
**TNM**				0.611
III	42 (24.00)	21 (28.77)	12 (30.00)	
IV	133 (76.00)	52 (71.23)	28 (70.00)	
**Family history**				0.56
No	132 (75.43)	58 (79.45)	33 (82.50)	
Yes	43 (24.57)	15 (20.55)	7 (17.50)	
**Tumor site**				0.913
Non-cardia cancer	77 (44.00)	30 (41.10)	17 (42.50)	
Cardia cancer	98 (56.00)	43 (58.90)	23 (57.50)	
**Tumor differentiation**				0.665
Medium to high	57 (32.57)	28 (38.36)	13 (32.50)	
Low	118 (67.43)	45 (61.64)	27 (67.50)	
**Treatment line**				0.104
1	109 (62.29)	48 (65.75)	32 (80.00)	
>1	66 (37.71)	25 (34.25)	8 (20.00)	
**Treatment regimen**				0.112
ICI	1 (0.57)	2 (2.74)	2 (5.00)	
ICI plus chemotherapy	128 (73.14)	55 (75.34)	26 (65.00)	
ICI plus targeted therapy	16 (9.14)	6 (8.22)	8 (20.00)	
ICI plus chemotherapy and targeted therapy	30 (17.14)	10 (13.70)	4 (10.00)	
**Number of metastases**				0.08
≤1	113 (64.57)	47 (64.38)	33 (82.50)	
>1	62 (35.43)	26 (35.62)	7 (17.50)	
**HER2**				0.16
Negative	81 (46.29)	30 (41.10)	26 (65.00)	
Positive	84 (48.00)	38 (52.05)	12 (30.00)	
Unknown	10 (5.71)	5 (6.85)	2 (5.00)	
**MMR**				0.224
Negative	144 (82.29)	57 (78.08)	28 (70.00)	
Positive	3 (1.71)	2 (2.74)	3 (7.50)	
Unknown	28 (16.00)	14 (19.18)	9 (22.50)	
**PD-L1**				0.255
Negative	41 (23.43)	23 (31.51)	9 (22.50)	
Positive	113 (64.57)	39 (53.42)	22 (55.00)	
Unknown	21 (12.00)	11 (15.07)	9 (22.50)	

IrAEs, immune-related adverse events; Uni-irAE, single-organ irAE; multi-irAEs, multi-organ irAEs; AGC, advanced gastric cancers; ECOG, Eastern Cooperative Oncology Group; TNM, tumor-node-metastasis; MMR, microsatellite mismatch repair HER2, human epidermal growth factor receptor-2; PD-L1, programmed cell death ligand 1.

The bold texts represents the grouping names for clinical characteristics.

The overall DCR of PD-1 treatment was 71.9% (288 patients), with 67.4% (118 patients) in the non-irAE group and 78.8% (89 patients) in the irAE group. As shown in **Table 2**, the DCR was significantly higher in the irAE group (*p*=0.037), uni-irAE group (*p*=0.019), and multi-irAE group (*p*=0.534) when compared with those of the non-irAE group, but no statistical difference existed between uni-irAE and multi-irAE group. These data demonstrated that the irAEs increased the DCR of AGC patients. Despite the multi-irAE group exhibiting a trend of ORR advantage, no statistical difference could be achieved.

**Table 2:** Response to ICI of irAEs, non-irAE, uni-irAE, and multi-irAE groups in AGC patients.

Comparison	PD	SD	PR	CR	DCR	*p*-value (DCR)	ORR	*p*-value (ORR)
**Non-irAE group vs. irAE group**						0.037		0.750
Non-irAE group	57	87	31	0	67.4%		17.7%	
irAE group	24	71	17	1	78.8%		15.9%	
**Uni-irAE group vs. multi-irAE group** Uni-irAE group Multi-irAE group	13 11	50 21	10 7	0 1	82.2% 72.5%	0.228	13.7% 20.0%	0.381
**Non-irAE group vs. uni-irAE group**						0.019		0.438
Non-irAE group	57	87	31	0	67.4%		17.7%	
Uni-irAE group	13	50	10	0	82.2%		13.7%	
**Non-irAE group vs. multi-irAE group**						0.534		0.735
Non-irAE group	57	87	31	0	67.4%		17.7%	
Multi-irAE group	11	21	7	1	72.5%		20.0%	

IrAEs, immune-related adverse events; Uni-irAE, single-organ irAE; Multi-irAEs, multi-organ irAEs; AGC, advanced gastric cancers; SD, stable disease; PR, partial response; PD, progressive disease; CR, complete response; ORR, objective response rate; DCR, disease control rate.

The bold texts represents the grouping names for clinical characteristics.

**Figure 1:**
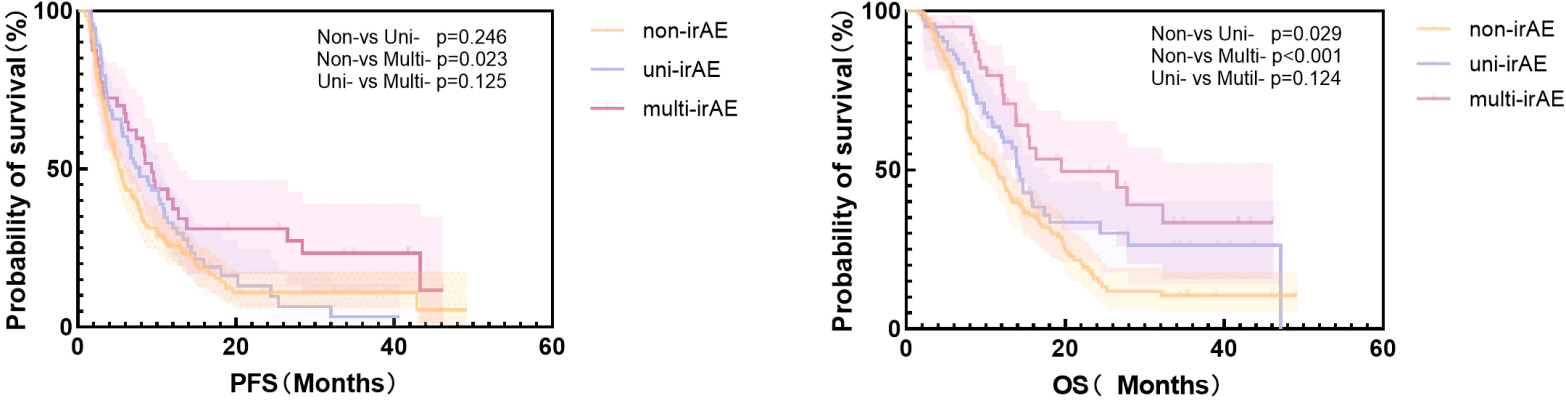
The Kaplan–Meier curve of progression-free survival (PFS) and overall survival (OS) of all patients.

The median PFS in the non-irAE, uni-irAE, and multi-irAEs group were 5.23 months (95% confidence interval [CI]: 4.42~6.05 months), 7.80 months (95% CI: 5.47~10.13 months), and 9.30 months (95% CI: 7.44~11.16 months), respectively (**Table 3**). The Kaplan–Meier curves of PFS and OS are presented in **Figure 1**, the multi-irAE group exhibited a substantial increase for median PFS (*p*=0.023) when compared with the non-irAE group, and no significant difference for PFS was identified between uni-irAE and multi-irAE groups (*p*=0.125), and no statistical difference between the non-irAE and uni-irAE groups was observed (*p*=0.246) either. The multivariate Cox proportional hazard model identified multi-irAEs as an independent prognostic factor for PFS (hazard ratio [HR]: 0.63, 95% CI: 0.41~0.96, *p*=0.031). Other characteristics such as age (HR 0.41, 95% CI 0.24~0.68, *p*<0.001 for those aged 45–65 years; HR 0.44, 95% CI 0.26~0.74, *p*=0.002 for those aged>65 years), ECOG score (HR 1.63, 95% CI 1.24~2.14, *p*<0.001), and treatment line (HR 1.87, 95% CI 1.40~2.48, *p*<0.001) were also identified as independent risk factors of PFS (**Table 4**). These data demonstrated that the occurrence of multi-irAEs was associated with better PFS.

**Table 3:** Median PFS and OS of non-irAE, uni-irAE, and multi-irAE groups in AGC patients.

Covariate	Median PFS (95% CI) (months)	*p-*value (Log-rank)	Median OS (95% CI) (months)	*p-*value (Log-rank)
**Non-irAE group vs. Uni-irAE group**		0.246		0.029
Non-irAE group	5.23 (4.42*~*6.05)		11.43 (9.16*~*13.70)	
Uni-irAE group	7.80 (5.47*~*10.13)		14.20 (13.14*~*15.26)	
**Non-irAE group vs. Multi-irAE group**		0.023		< 0.001
Non-irAE group	5.23 (4.42*~*6.05)		11.43 (9.16*~*13.70)	
Multi-irAE group	9.30 (7.44*~*11.16)		19.50 (3.75*~*35.25)	
**Uni-irAE group vs. Multi-irAE group**		0.125		0.124
Uni-irAE group	5.23 (4.42*~*6.05)		14.20 (13.14*~*15.26)	
Multi-irAE group	9.30 (7.44*~*11.16)		19.50 (3.75*~*35.25)	

IrAEs, immune-related adverse events; Uni-irAE, single-organ irAE; Multi-irAEs, multi-organ irAEs; AGC, advanced gastric cancers; OS, overall survival; PFS, progression-free survival.

The bold texts represents the grouping names for clinical characteristics.

**Table 4:** Univariate and multiple Cox proportional hazard models on PFS in AGC patients.

Covariate	Univariate analysis			Multivariate analysis		
	HR	95% CI	*p-*value	HR	95% CI	*p-*value
Gender						
Female	1.00 (reference)					
Male	0.85	0.63~1.14	0.278			
Age						
18-44	1.00 (reference)			1.00 (reference)		
45-65	0.53	0.32~0.87	0.013	0.41	0.24~0.68	<.001
>65	0.49	0.30~0.81	0.006	0.44	0.26~0.74	0.002
ECOG						
≤1	1.00 (reference)			1.00 (reference)		
>1	1.71	1.31~2.22	<.001	1.63	1.24~2.14	<.001
TNM						
III	1.00 (reference)			1.00 (reference)		
IV	1.51	1.11~2.04	0.008	1.29	0.94~1.76	0.120
Family history						
No	1.00 (reference)					
Yes	1.02	0.75~1.39	0.875			
Tumor site						
Non-cardia cancer	1.00 (reference)					
Cardia cancer	1.03	0.79~1.33	0.846			
Tumor differentiation						
Medium to high	1.00 (reference)					
Low	1.00	0.76~1.31	0.982			
Treatment line						
1	1.00 (reference)			1.00 (reference)		
>1	1.90	1.46~2.48	<.001	1.87	1.40~2.48	<.001
Treatment regimen						
I	1.00 (reference)					
I+C	1.56	0.50~4.89	0.446			
I+T	2.93	0.89~9.67	0.077			
I+C+T	2.39	0.74~7.73	0.147			
Number of metastases						
≤1	1.00 (reference)					
>1	1.17	0.89~1.53	0.267			
HER2						
Negative	1.00 (reference)			1.00 (reference)		
Positive	0.76	0.58~0.99	0.046	0.80	0.60~1.05	0.108
Unknown	0.62	0.35~1.09	0.099	0.69	0.35~1.33	0.265
MMR						
Negative	1.00 (reference)			1.00 (reference)		
Positive	0.35	0.13~0.95	0.038	0.47	0.17~1.31	0.150
Unknown	1.00	0.72~1.40	0.989	1.04	0.69~1.56	0.861
PD-L1						
Negative	1.00 (reference)			1.00 (reference)		
Positive	0.70	0.52~0.95	0.021	0.74	0.54~1.03	0.074
Unknown	0.89	0.59~1.35	0.578	0.91	0.55~1.50	0.701
irAE						
Non-	1.00 (reference)			1.00 (reference)		
Uni-	0.84	0.62~1.13	0.248	0.86	0.63~1.17	0.324
Mul-	0.60	0.40~0.91	0.015	0.63	0.41~0.96	0.031

IrAEs, immune-related adverse events; Uni-irAE, single-organ irAE; Multi-irAEs, multi-organ irAEs; AGC, advanced gastric cancer; HR, hazard ratio; CI, confidence interval; PFS, progression-free survival; ECOG, Eastern Cooperative Oncology Group; TNM, tumor-node-metastasis; MMR, microsatellite mismatch repair HER2, human epidermal growth factor receptor-2; PD-L1, programmed cell death ligand 1.

The median OS in the non-irAE, uni-irAE, and multi-irAE groups were 11.43 months (95% CI: 9.16~13.70 months), 14.20 months (95% CI: 13.14~15.26 months), and 19.50 months (95% CI: 3.75~35.25 months), respectively (**Table 3**). The median OS of multi-irAEs displayed a significant extension compared with that of the non-irAE group (*p*<0.001); the uni-irAE group also showed an extended median OS than that of the non-irAE group (*p*=0.029), but no significant difference existed between the uni-irAE and multi-irAE groups (*p*=0.124). The multivariate Cox proportional hazard model indicated that multi-irAEs was an independent prognostic factor for OS (HR 0.47, 95% CI 0.29~0.76, *p*=0.002). In addition, age (HR 0.41, 95% CI 0.24~0. 71, *p*=0.001 for those aged 45–65 years, HR 0.47, 95% CI 0.27~0.80, *p*=0.006 for those aged >65 years), ECOG score (HR 1.57, 95% CI 1.18~2.11, *p*=0.002), TNM stage (HR 1.46, 95% CI 1.01~2.11, *p*=0.043), and treatment line (HR 1.74, 95% CI 1.30~2.34, *p*< 0.001) remained as independent prognostic factors for OS (**Table 5**). All these results indicated that multi-irAEs modify the treatment efficiency of ICI by increasing DCR, PFS, and OS.

**Table 5:** Univariate and multiple Cox proportional hazard model on OS in AGC patients.

Covariate	Univariate analysis			Multivariate analysis		
	HR	95% CI	*p-*value	HR	95% CI	*p-*value
Gender						
Female	1.00 (reference)					
Male	0.98	0.71~1.35	0.885			
Age						
18-44	1.00 (reference)			1.00 (reference)		
45-65	0.47	0.28~0.79	0.004	0.41	0.24~0.71	0.001
>65	0.47	0.28~0.79	0.005	0.47	0.27~0.80	0.006
ECOG						
≤ 1	1.00 (reference)			1.00 (reference)		
>1	1.66	1.25~2.21	<.001	1.57	1.18~2.11	0.002
TNM						
III	1.00 (reference)			1.00 (reference)		
IV	1.7	1.19~2.42	0.003	1.46	1.01~2.11	0.043
Family history						
No	1.00 (reference)					
Yes	1.04	0.74~1.45	0.842			
Tumor site						
Non-cardia cancer	1.00 (reference)					
Cardia cancer	0.92	0.69~1.23	0.583			
Tumor differentiation						
Medium to high	1.00 (reference)					
Low	1.02	0.76~1.39	0.874			
Treatment line						
1	1.00 (reference)			1.00 (reference)		
>1	1.9	1.42~2.53	<.001	1.74	1.30~2.34	<.001
Treatment regimen						
I	1.00 (reference)					
I+C	0.98	0.31~3.08	0.972			
I+T	1.49	0.45~4.95	0.516			
I+C+T	1.45	0.45~4.75	0.535			
Number of metastases						
≤1	1.00 (reference)					
>1	1.13	0.84~1.52	0.426			
HER2						
Negative	1.00 (reference)					
Positive	0.79	0.59~1.06	0.117			
Unknown	0.72	0.39~1.32	0.284			
MMR						
Negative	1.00 (reference)					
Positive	0.52	0.19~1.41	0.199			
Unknown	0.91	0.63~1.32	0.631			
PD-L1						
Negative	1.00 (reference)					
Positive	0.84	0.60~1.17	0.301			
Unknown	0.84	0.53~1.33	0.456			
irAE						
Non-	1.00 (reference)			1.00 (reference)		
Uni-	0.68	0.48~0.96	0.027	0.76	0.53~1.07	0.117
Mul-	0.43	0.27~0.70	<.001	0.47	0.29~0.76	0.002

IrAEs, immune-related adverse events; Uni-irAE, single-organ irAE; Multi-irAEs, multi-organ irAEs; AGC, advanced gastric cancer; HR, hazard ratio; CI, confidence interval; OS, overall survival; ECOG, Eastern Cooperative Oncology Group; TNM, tumor-node-metastasis; MMR, microsatellite mismatch repair HER2, human epidermal growth factor receptor-2; PD-L1, programmed cell death ligand 1.

### Co-occurrence pattern analysis for multi-irAEs

The most common irAEs in the uni-irAE group was hypothyroidism or hyperthyroidism (27.40%), whereas the common types of co-occurrence multi-irAEs were skin plus adrenal gland (15.00%), thyroid plus adrenal gland (12.50%), and thyroid plus heart (12.50%) (**Supplementary Table S2**). Among the multi-irAE group, 14 cases (35.00%) co-occurred simultaneously, whereas the remaining occurred sequentially. Univariate logistic regression analyses were performed to evaluate the co-occurrence pattern of multi-irAEs (**Table 6**), and the results showed that the thyroid, adrenal gland, heart, skin, and lung irAEs were more likely to develop multi-irAEs (odds ratio [OR] 2.40, 95% CI 1.07~5.37, *p*=0.033 for thyroid irAEs; OR 3.08, 95% CI 1.29~7.36, *p*=0.011 for adrenal gland irAEs; OR 3.72, 95% CI 1.24~11.18, *p*=0.019 for heart irAEs; OR 2.43, 95% CI 1.06~5.58, *p*=0.035 for skin irAEs; OR 4.31, 95% CI 1.21~15.38, *p*=0.024 for lung irAEs). The remaining gastrointestinal system, hematological system, liver, kidney, and musculoskeletal system irAEs are not related to the occurrence of multi-irAEs (all *p*>0.05).

**Table 6:** Univariate logistic regression analysis of the relationship between each organ-specific irAE and multi-irAEs in AGC patients.

Covariate	OR(95% CI)	*p-*value
Thyroid	2.40 (1.07~5.37)	0.033
Adrenal gland	3.08 (1.29~7.36)	0.011
Heart	3.72 (1.24~11.18)	0.019
Gastrointestinal system	1.40 (0.30~6.59)	0.671
Skin	2.43 (1.06~5.58)	0.035
Lung	4.31 (1.21~15.38)	0.024
Liver	1.92 (0.45~8.12)	0.377
Kidney	0.00 (0.00~Inf)	0.988
Hematological system	1.87 (0.25~13.79)	0.540
Musculoskeletal system	5.84 (0.59~58.09)	0.132

IrAEs, immune-related adverse events; Multi-irAEs, multi-organ irAEs; AGC, advanced gastric cancer; OR, odds ratio.

The association between patients’ characteristics and multi-irAEs was performed by univariate and multivariate logistic regression analyses, and only those who were HER2 positive were less likely to experience multi-irAEs (OR 0.39, 95% CI 0.17~0.92, *p*=0.031) (**Supplementary Table S3**).

### Timepoint of irAE onset

**Figure 2:**
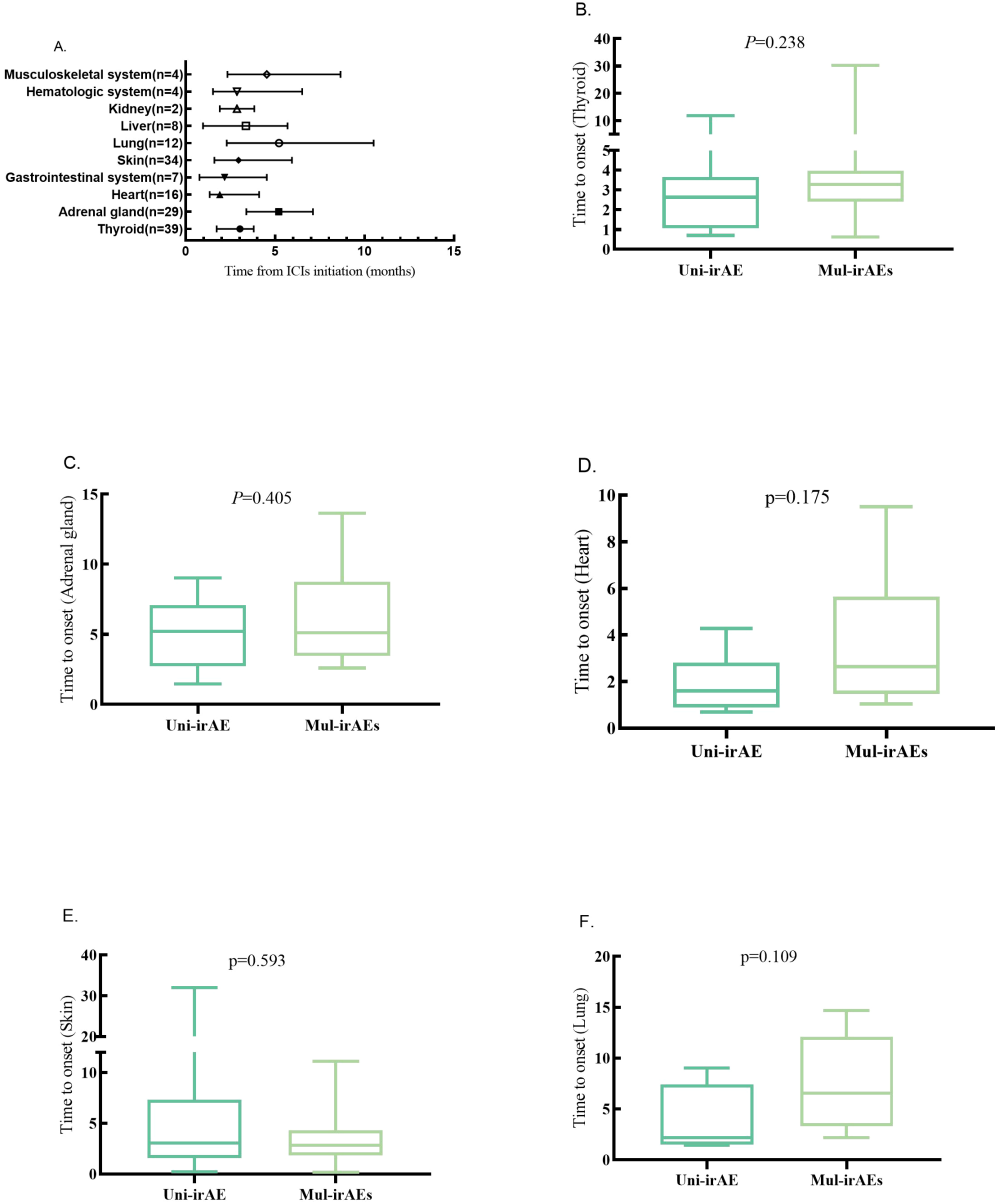
**(A)** Time to onset of one-specific organ irAEs (median, range, and months). Time to onset of thyroid **(B)**, adrenal gland **(C)**, heart **(D)**, skin **(E)**, and lung **(F)** irAEs in uni-irAE and multi-irAE groups.

The onset time of each irAE is shown in **Figure 2**; the median time points of the five irAEs associated with co-occurrence of multi-irAEs were 3.03 months for thyroid irAE, 5.20 months for adrenal gland irAE, 1.90 months for heart irAE, 2.95 months for skin irAE, and 5.22 months for lung irAE. The onset of heart irAE with high morbidity and mortality was the mostly earliest among these all types. The median occurrence timepoint of the same irAE between uni-irAE and multi-irAEs groups was compared, and no statistical difference for each irAE between the two groups (**Figure 2**). These data implied that the time extension was not associated with the occurrence of multi-irAEs.

### The impact of each organ-specific irAE on ICI treatment

We performed the treatment efficacy and prognosis analysis for all organ-specific irAEs. The thyroid irAE displayed higher DCR than that of the other group (87.2% vs. 69.5%, *p*=0.022), but no association with DCR or ORR could be found for the remaining organ-specific irAEs (**Supplementary Table S4**). As shown in **Supplementary Table S5** and **Supplementary Figure S1**, only the thyroid and adrenal gland irAE exhibited a significant increase for median PFS when compared individually with the others group (thyroid: 11.37 months [95% CI: 7.20~15.54] vs. 5.77 months [95% CI: 4.81~6.73], *p*=0.023; adrenal gland: 10.23 months [95% CI: 8.26~12.21] vs. 5.83 months [95% CI: 4.92~6.75], *p*=0.006) by the Kaplan–Meier method with the log-rank test. The multivariate survival analysis indicated that the thyroid and adrenal gland irAEs were independent risk factors for extending PFS (thyroid: HR 0.65, 95% CI 0.44~0.96, *p*=0.031; adrenal gland: HR 0.50, 95% CI 0.30~0.83, *p*=0.008) (**Supplementary Tables S8**, **S9**). Furthermore, the thyroid, adrenal gland, and skin irAE patients exhibited a substantial increase for median OS comparing with the other groups (thyroid: 24.43 months [95% CI: 9.61~39.25] vs. 12.27 months [95% CI: 10.75~13.78], *p*=0.004; adrenal gland: 16.30 months [95% CI: 14.70~NA] vs. 12.27 months [95% CI: 11.05~13.48], *p*=0.003; skin: 17.37 months [95% CI: 14.36~20.37] vs. 12.40 months [95% CI: 10.99~13.81], *p*=0.016) (**Supplementary Table S5**, **Supplementary Figure S1**). The multivariate survival analysis indicated that the thyroid, adrenal gland, and skin irAEs were independent risk factors for extending OS (thyroid: HR 0.53, 95% CI 0.34~0.84, *p*=0.007; adrenal gland: HR 0.47, 95% CI 0.26~0.85, *p*=0.012; skin: HR 0.58, 95% CI 0.35~0.97, *p*= 0.039) (**Supplementary Tables S10**-**S12**). No statistically significant impact on PFS or OS was observed for heart and lung irAEs (all *p*>0.05) (**Supplementary Tables S6**, **S7**).

## Discussion

The occurrence of irAEs seemed to be a good predictor for ICI treatment in many cancer types including GC patients (25, 27, 38, 39). The occurrence of irAEs is linked with better DCR and prolonged OS and PFS in AGC patients with nivolumab monotherapy; we also found that irAEs were associated with prolonged PFS in AGC patients, but few studies focus on the association of uni-irAE or multi-irAEs with the outcome of AGC patients (27, 40, 41). In the present study, the multivariate analysis proved the association with both PFS and OS for multi-irAE AGC patients. Although the PFS extension of uni-irAE did not show statistical difference probably due to the small sample size, it displayed both the better DCR and the extension of OS in univariate analysis compared with the non-irAE group. Multiple centers with larger sample sizes are needed to confirm whether it is truly unrelated to PFS and OS. We also used the least absolute shrinkage and selection operator (LASSO) regression model suitable for small sample size analysis to verify these results. Notably, the results derived from these models aligned consistently with our initial findings referring to the association between multi-irAE and treatment outcomes (data not shown). These results are consistent with the results from the advanced renal cell carcinoma and SCLC, which suggest that the occurrence of irAEs indicates better survival, and multi-irAE patients exhibited an improved survival time than those of uni-irAE or non-irAE patients (34).

The precise mechanism underlying the enhanced efficacy of ICIs in patients with irAEs remains unclear, and few discussions regarding to the mechanism by which treatment efficacy of multi-irAEs is superior to uni-irAE. Functional experiments based on irAE-related experimental animals need to be performed to reveal the corresponding changes in the tumor microenvironment, including activated CD8, mature B cells, and even mature tertiary lymphoid structures, Treg cells, tumor associated macrophages, cytokines, etc.

One hypothesis for irAE-mediated prognosis posits that ICIs reactivate exhausted T cells with cross-reactivity to both tumor and normal tissue antigens, resulting in enhanced antitumor immunity and irAEs (42). However, some scholars also argue that the related immune response induced by ICIs may be non-specific, so it may attack both tumor and non-tumor cells indiscriminately (43). Another possible explanation is that the inflammatory cytokine involved in the occurrence of irAEs could modify the treatment efficiency in cancer patients, and the patients carrying genetic variations near the interleukin-7 (IL-7) gene may have both a higher incidence of irAEs and improved survival. The abnormal expression of IL-7 might increase the stability of lymphocytes as well as block PD-1 to create a more favorable microenvironment corresponding to the ICI treatment (44, 45). Another cytokine of interferon gamma (IFN-γ) also associated with both the occurrence of irAEs and better survival expectation (46–48). We had measured the blood cytokine levels of interleukin-2 (IL-2), interleukin-4 (IL-4), interleukin-6 (IL-6), interleukin-10 (IL-10), IFN-γ, and tumor necrosis factor alpha (TNF-α) between patients with and without irAEs, but no statistically significant differences were found (data not shown). We need to check other cytokines in the future. Thirdly, the gene mutations and expressing changes might be implicated in the occurrence of irAEs (44, 49); we conducted an expression profiling analysis for blood microRNAs (miRNAs) including 16 miRNAs, which were proved to modify the AGC outcome with ICI treatment, but no candidate miRNA could be identified for their association with irAE occurrence (data not shown). Fourthly, the gut microbiota species and their metabolic pathway are involved in the occurrence of irAEs in GC patients; the gut microbiota species were also proved to enhance the treatment efficiency of ICI (50–52), so there is every chance that gut microbiota can mediate the irAE occurrence as well as modify the treatment efficiency. According to the above conjecture, the occurrence of irAEs and antitumor immunity may share common pathways to complete the immune response, which may involve various changes of the immune microenvironment upon treatment (26, 53). Therefore, we speculated that the multi-irAEs indicated a stronger immune response, or it induced a more altered immune microenvironment to better adapt to immunotherapy.

We conducted separate analyses of irAEs for each organ to elucidate the co-occurrence patterns, timing of onset, and impact on prognosis. To the best of our knowledge, our study represents the first comprehensive and detailed analysis of irAEs for each organ in AGC. We did not find correlations between the multi-irAEs and clinical characteristics with good prognosis prediction, such as age, ECOG score, and treatment lines. However, we found that heart, lung, skin, thyroid, and adrenal gland irAEs were associated with the co-occurrence of multi-organ irAEs. A previous retrospective study also revealed that skin irAEs and thyroid irAEs were associated with the co-occurrence of multi-irAEs in patients among multiple tumor types (34). According to our research data, it seems that thyroid plus adrenal gland irAE, thyroid plus heart irAE, thyroid plus skin irAE, or skin plus adrenal gland irAE are more common co-occurring combinations. One possible explanation for the co-occurrence of irAEs is that the adaptive immune system eliminates most of the high-affinity self-reactive cells (immune cells that can recognize and attack self-antigens) through a complex selection process. The leaving out of self-reactive cells after immune clearance will be activated during immunotherapy, and some overlooked immune cells may express different antibodies against self-antigens and attack the same self-antigens expressed in the different organs, thereby inducing the co-occurrence of irAEs (54). The anti-thyroglobulin IgG increased during the liver irAE, which can also initiate the thyroid irAE (8, 55). Another potential explanation is that ICI disrupts peripheral immune tolerance, thereby enabling immune cells to re-recognize specific harmless microorganisms or environmental proteins, potentially leading to irAEs in barrier organs such as skin and lungs (54). The true mechanism of co-occurrence of multi-irAEs is still controversial.

The onset of heart irAE with high morbidity and mortality was the mostly earliest among these irAEs, which may imply that early-onset irAEs might be more severe (56). Additionally, as the symptoms of skin irAEs can be easily identified without the need for laboratory tests, its onset may manifest at an earlier stage. However, there was no significant difference in the onset time of heart, lung, skin, thyroid, and adrenal gland irAEs between uni-irAE and multi-irAEs groups, which means that the occurrence of multi-irAE is unrelated to time. This is consistent with Yamaguchi’s data in multiple tumor types (34).

As for the association of specific organ irAEs and the outcome of these AGC patients, we found that the thyroid and adrenal gland irAEs predict a longer survival period referring to PFS, whereas the thyroid, adrenal gland, and skin irAEs are associated with longer OS. Previous research also confirmed our finding regarding the thyroid, adrenal gland, and skin irAEs for their association with better treatment outcome in AGC and other cancer patients (57). They believed that skin, thyroid, and adrenal gland irAE were indicative of a robust immune response for the antitumor efficacy of ICI.

The possible reasons for the improvement in prognosis associated with skin irAEs are as follows: The skin is abundant of immune cells with the tissue-resident memory CD8 T cells being the primary type, which can initiate the immunologic reaction to eliminate tumor. In addition, the sensory nerve cell in the skin can secrete certain neuropeptides (such as substance P) to drive the expression of proinflammatory cytokines, thereby enhancing the responsiveness of immune cells for tumor clearance (58, 59). Although the specific pathways underlying irAEs in the endocrine system are not yet fully understood, potential mechanisms may involve enhanced T-cell activation, autoantibody stimulation, and increased cytokine levels (57). The thyroid irAE-related genetic variations, which were identified by whole-genome sequencing in ICI-treated cancer patients, could modify the systemic immune response to PD-1 blockade (60). Additionally, thyroid and adrenal gland irAEs are generally less severe without intermittent treatment of ICI (61). Conversely, irAEs affecting heart, lungs, liver, and muscles often present with more severe symptoms or chronic persistence that need a large amount of glucocorticoids to suppress the immune response with the intermittent treatment of ICI. This may partially explain why thyroid and adrenal gland irAE achieved better outcomes. The relationship between gastrointestinal system, lung, renal, and liver irAEs and prognosis are still controversial and mixed. Further investigation with larger sample sizes is needed in the future.

Given the above, these results highlight the clinical importance of identifying co-occurrence patterns of irAEs and support the specific monitoring, diagnosing, and managing for skin and endocrine irAEs, because they can be served as prognostic biomarkers for multi-organ irAEs.

This study has some limitations: 1. It was a retrospective observational cohort study performed at a single center with a relatively small size. Although the overall number of patients has basically met the requirement, the sample of patients experiencing single-organ irAEs was still small, although not for multi-organ irAEs. 2. Selection biases such as differences in patient treatment protocols and variations in lines of therapy might influence the final results. 3. The lack of diversity in the patient population. 4. A short follow-up period is also a limitation. However, irAE was originally an exclusionary diagnosis without definitive pathological evidence to rely on; more and more irAEs were recognized with the increasingly widespread application of immunotherapy, which may be one of the reasons for the relatively changed results referring to the impact of irAEs on ICI treatment efficiency. In the future, we need to carry out a larger sample size study with multicenter and multi-tumor species to further verify the value of irAEs in ICI treatment.

## Conclusion

Multi-organ irAEs can be used as a predictive indicator for good efficacy in AGC patients with ICI treatment. The thyroid, adrenal gland, heart, skin, and pulmonary irAEs are often accompanied by the multi-organ irAE occurrence. The organ-specific irAEs for thyroid, adrenal gland, and skin were associated with better outcome. Early assessment of irAEs is crucial for patients for enhancing early monitoring and early preventive interventions.

## Data Availability

The original contributions presented in the study are included in the article/**Supplementary Material**. Further inquiries can be directed to the corresponding authors.

## Supplementary material

Supplementary Table 1 Treatment regimen of AGC patients.

Supplementary Table 2 The number of irAE cases and the timing of onset for uni-irAE and multi-irAEs groups in AGC patients.

Supplementary Table 3 Univariate and multivariate logistic regression analyses for the clinical characteristics associated with the occurrence of multi-irAEs in AGC patients.

Supplementary Table 4 Response rate analysis of each organ-specific irAE vs. other patients in AGC patients.

Supplementary Table 5 Median PFS and OS of each organ-specific group vs. other patients in AGC patients.

Supplementary Table 6 Univariate analysis for the clinical characteristics associated with PFS and OS in AGC patients (each organ-specific irAE with a case number of more than 10 patients was included in clinical characteristics for analysis).

Supplementary Table 7 Univariate analysis for the clinical characteristics associated with PFS and OS in AGC patients (each organ-specific irAE with a case number of more than 10 patients was included in clinical characteristics for analysis).

Supplementary Table 8 Multivariate Cox proportional hazard model analysis for the association between PFS and organ-specific irAEs in AGC patients.

Supplementary Table 9 Multivariate Cox proportional hazard model analysis for the association between PFS and organ-specific irAEs in AGC patients.

Supplementary Table 10 Multivariate Cox proportional hazard model analysis for the association between OS and organ-specific irAEs in AGC patients.

Supplementary Table 11 Multivariate Cox proportional hazard model analysis for the association between OS and organ-specific irAEs in AGC patients.

Supplementary Table 12 Multivariate Cox proportional hazard model analysis for the association between OS and organ-specific irAEs in AGC patients.

Supplementary Figure 1
**(A.-J)** The Kaplan–Meier curve of progression-free survival (PFS) and overall survival (OS) of each specific irAE with a case number of more than 10 patients in AGC patients. **(A)** PFS of thyroid irAE patients. **(B)** OS of thyroid irAE patients. **(C)** PFS of adrenal gland irAE patients. **(D)** OS of adrenal gland irAE patients. E: PFS of heart irAE patients. **(F)** OS of heart irAE patients. **(G)** PFS of skin irAE patients. **(H)** OS of skin irAE patients. I: PFS of lung irAE patients. **(J)** OS of lung irAE patients.
