# Supplementary material for: The impact of immune-related adverse events on the outcome of advanced gastric cancer patients with immune checkpoint inhibitor treatment
Source: Front Immunol. 2024 Dec 24;15:1503316. doi: 10.3389/fimmu.2024.1503316 (PMC11703953; doi:10.3389/fimmu.2024.1503316)
Supplement: Supplementary Table 1 — Treatment regimen of AGC patients. [file DataSheet1.docx]

**Table S1.** Treatment regimen of AGC patients.

| Number of patients | Therapeutic regimen | | |
| --- | --- | --- | --- |
|  | ICIs | Targeted drug | Chemotherapeutic regimen |
| 2 | Pembrolizumab | Apatinib | - |
| 2 | Pembrolizumab | Lenvatinib | - |
| 4 | Pembrolizumab | - | Oxaliplatin/Lobaplatin+S-1 |
| 2 | Pembrolizumab | - | Oxaliplatin+Capecitabine |
| 3 | Pembrolizumab | - | Paclitaxel |
| 1 | Pembrolizumab |  | Paclitaxel+5-FU |
| 2 | Pembrolizumab |  | Paclitaxel+S-1 |
| 1 | Pembrolizumab | Trastuzumab | Paclitaxel |
| 1 | Pembrolizumab | Apatinib | Paclitaxel |
| 1 | Pembrolizumab | Trastuzumab | Oxaliplatin+S-1 |
| 1 | Pembrolizumab | Trastuzumab | Oxaliplatin+Capecitabine |
| 2 | Camrelizumab | - | - |
| 11 | Camrelizumab | - | Oxaliplatin/Lobaplatin+S-1 |
| 13 | Camrelizumab | - | Oxaliplatin+Capecitabine |
| 3 | Camrelizumab | - | Oxaliplatin+Paclitaxel |
| 1 | Camrelizumab | - | Cisplatin+5-FU |
| 1 | Camrelizumab | - | Irinotecan |
| 1 | Camrelizumab | - | Paclitaxel |
| 4 | Camrelizumab | - | Paclitaxel+S-1 |
| 1 | Camrelizumab | - | Paclitaxel+S-1+Oxaliplatin |
| 12 | Camrelizumab | Apatinib | - |
| 2 | Camrelizumab | Lenvatinib | - |
| 6 | Camrelizumab | Apatinib | Paclitaxel |
| 1 | Camrelizumab | Apatinib | S-1 |
| 2 | Camrelizumab | Trastuzumab | Paclitaxel |
| 1 | Camrelizumab | Trastuzumab | Irinotecan |
| 1 | Camrelizumab | Trastuzumab | Oxaliplatin+S-1 |
| 2 | Sintilimab | - | - |
| 44 | Sintilimab | - | Oxaliplatin/Lobaplatin/Cisplatin+S-1 |
| 4 | Sintilimab | - | Oxaliplatin+5-FU |
| 24 | Sintilimab | - | Oxaliplatin+Capecitabine |
| 4 | Sintilimab | - | Oxaliplatin+Paclitaxel |
| 4 | Sintilimab | - | Irinotecan |
| 4 | Sintilimab | - | Paclitaxel |
| 2 | Sintilimab | - | Paclitaxel+5-FU |
| 12 | Sintilimab | - | Paclitaxel+S-1 |
| 2 | Sintilimab |  | Paclitaxel+Nedaplatin/Oxaliplatin |
| 4 | Sintilimab | Apatinib | - |
| 1 | Sintilimab | Apatinib | Oxaliplatin |
| 5 | Sintilimab | Apatinib | Irinotecan |
| 2 | Sintilimab | Apatinib | Paclitaxel+S-1 |
| 1 | Sintilimab | Apatinib | Paclitaxel+Lobaplatin |
| 1 | Sintilimab | Trastuzumab | S-1 |
| 4 | Sintilimab | Trastuzumab | Oxaliplatin+S-1 |
| 3 | Sintilimab | Trastuzumab | Oxaliplatin+Capecitabine |
| 2 | Sintilimab | Trastuzumab | Paclitaxel |
| 2 | Tislelizumab | - | 5-FU |

**Table S1. continued.**

| 4 | Tislelizumab | - | S-1 |
| --- | --- | --- | --- |
| 14 | Tislelizumab | - | Oxaliplatin/Lobaplatin+S-1 |
| 1 | Tislelizumab | - | Paclitaxel+S-1+Oxaliplatin |
| 8 | Tislelizumab | - | Oxaliplatin+Capecitabine |
| 2 | Tislelizumab | - | Capecitabine |
| 1 | Tislelizumab | - | Irinotecan+Capecitabine |
| 2 | Tislelizumab | - | Irinotecan+S-1 |
| 2 | Tislelizumab | - | Paclitaxel |
| 5 | Tislelizumab | - | Paclitaxel+S-1 |
| 2 | Tislelizumab | - | Oxaliplatin/Lobaplatin+Paclitaxel |
| 3 | Tislelizumab | Apatinib | - |
| 1 | Tislelizumab | Trastuzumab | - |
| 1 | Tislelizumab | Lenvatinib | - |
| 1 | Tislelizumab | Disitamab Vedotin | - |
| 3 | Tislelizumab | Apatinib | Irinotecan+Capecitabine |
| 1 | Tislelizumab |  | Irinotecan |
| 1 | Tislelizumab |  | Oxaliplatin+S-1 |
| 1 | Tislelizumab | Trastuzumab | Capecitabine |
| 1 | Tislelizumab | Trastuzumab | Irinotecan+Capecitabine |
| 1 | Tislelizumab | Trastuzumab | Irinotecan |
| 1 | Tislelizumab | Trastuzumab | Paclitaxel+Capecitabine |
| 1 | Tislelizumab | Trastuzumab | Oxaliplatin+Capecitabine |
| 1 | Tislelizumab | Trastuzumab | S-1 |
| 1 | Tislelizumab | Trastuzumab | Paclitaxel |
| 1 | Nivolumab | - | Oxaliplatin+5-FU |
| 4 | Nivolumab | - | Oxaliplatin+S-1 |
| 3 | Nivolumab | - | Oxaliplatin+Capecitabine |
| 2 | Nivolumab | - | Paclitaxel |
| 1 | Nivolumab | - | Paclitaxel+S-1 |
| 2 | Serplulimab | - | Paclitaxel |
| 1 | Toripalimab | - | - |
| 2 | Toripalimab | Apatinib | - |
| 2 | Toripalimab | - | Oxaliplatin+Capecitabine |
| 1 | Toripalimab | - | Oxaliplatin+S-1 |
| 1 | Toripalimab | - | S-1 |

AGC, advanced gastrointestinal cancers; ICIs, immune checkpoint inhibitors.

**Table S2.** The number of irAE cases and the timing of onset for uni-irAE and multi-irAEs groups in AGC patients.

| **Covariate** | **Time (months)** | **N (number)** | **% (percentage)** |
| --- | --- | --- | --- |
| **Uni-irAE** |  |  |  |
| Thyroid | 2.63 | 20 | 27.40 |
| Adrenal gland | 5.20 | 13 | 18.81 |
| Heart | 1.60 | 6 | 8.22 |
| Gastrointestinal system | 2.00 | 4 | 9.59 |
| Skin | 3.07 | 17 | 2.33 |
| Lung | 2.17 | 4 | 5.48 |
| Liver | 5.18 | 4 | 5.48 |
| Kidney | 2.87 | 2 | 2.74 |
| Hematologic system | 1.95 | 2 | 2.74 |
| Musculoskeletal system | 3.23 | 1 | 1.37 |
| **Multi-irAEs** |  |  |  |
| Thyroid+ Adrenal gland | 3.80 | 5 | 12.5 |
| Thyroid+ Heart | 3.60 | 5 | 12.5 |
| Thyroid+ Skin | 2.63 | 3 | 7.50 |
| Thyroid+ Hematologic system | 7.70 | 1 | 2.50 |
| Thyroid+ Lung | 30.27 | 1 | 2.50 |
| Thyroid+ Musculoskeletal system | 15.23 | 1 | 2.50 |
| Skin+ Adrenal gland | 5.42 | 6 | 15.00 |
| Skin+ Lung | 3.75 | 2 | 5.00 |
| Skin+ Gastrointestinal system | 5.70 | 3 | 7.50 |
| Skin+ Liver | 2.17 | 2 | 5.00 |
| Skin+ Heart | 3.30 | 1 | 2.50 |
| Adrenal gland+ Lung | 14.67 | 1 | 2.50 |
| Heart+ Musculoskeletal system | 2.03 | 1 | 2.50 |
| Heart+ Hematologic system | 2.93 | 1 | 2.50 |
| Liver+ Lung | 7.37 | 1 | 2.50 |
| Liver+ Heart | 1.77 | 1 | 2.50 |
| Lung+ hyperglycemia | 3.13 | 1 | 2.50 |
| Thyroid+ Skin+ Heart | 8.47 | 1 | 2.50 |
| Thyroid+ Adrenal gland+ Lung | 11 | 1 | 2.50 |
| Thyroid+ Adrenal gland+ hyperglycemia | 8.77 | 1 | 2.50 |
| Lung+ Musculoskeletal system+ Adrenal gland | 5.83 | 1 | 2.50 |

IrAEs, immune-related adverse events; AGC, advanced gastric cancer; Uni-irAE, single-organ irAE; Multi-irAEs, multi-organ irAEs.

**Table S3.** Univariate and multivariate logistic regression analyses for the clinical characteristics associated with the occurrence of multi-irAEs in AGC patients.

| **Covariate** | **Univariate Analysis** | | |  | **Multivariate Analysis** | | |
| --- | --- | --- | --- | --- | --- | --- | --- |
|  | **OR** | **95% CI** | ***p-*value** |  | **OR** | **95% CI** | ***p-*value** |
| **Gender** |  |  |  |  |  |  |  |
| Female | 1.00 (Reference) |  |  |  |  |  |  |
| Male | 1.79 | 0.74 ~ 4.35 | 0.196 |  |  |  |  |
| **Age** |  |  |  |  |  |  |  |
| 18-44 | 1.00 (Reference) |  |  |  |  |  |  |
| 45-65 | 0.73 | 0.11 ~ 4.75 | 0.743 |  |  |  |  |
| ＞65 | 0.92 | 0.14 ~ 6.01 | 0.743 |  |  |  |  |
| **ECOG** |  |  |  |  |  |  |  |
| ≤1 | 1.00 (Reference) |  |  |  |  |  |  |
| ＞1 | 1.02 | 0.46 ~ 2.27 | 0.957 |  |  |  |  |
| **TNM** |  |  |  |  |  |  |  |
| Ⅲ | 1.00 (Reference) |  |  |  |  |  |  |
| Ⅳ | 0.94 | 0.40 ~ 2.19 | 0.890 |  |  |  |  |
| **Family history** |  |  |  |  |  |  |  |
| No | 1.00 (Reference) |  |  |  |  |  |  |
| Yes | 0.82 | 0.30 ~ 2.22 | 0.696 |  |  |  |  |
| **Tumor site** |  |  |  |  |  |  |  |
| Non-cardia cancer | 1.00 (Reference) |  |  |  |  |  |  |
| Cardia cancer | 0.94 | 0.43 ~ 2.06 | 0.885 |  |  |  |  |
| **Tumor differentiation** |  |  |  |  |  |  |  |
| Medium to high | 1.00 (Reference) |  |  |  |  |  |  |
| Low | 1.29 | 0.77 ~ 1.32 | 0.970 |  |  |  |  |
| **Treatment line** |  |  |  |  |  |  |  |
| 1 | 1.00 (Reference) |  |  |  |  |  |  |
| >1 | 1.90 | 0.57 ~ 2.91 | 0.536 |  |  |  |  |
| **Treatment regimen** |  |  |  |  |  |  |  |
| I | 1.00 (Reference) |  |  |  |  |  |  |
| I+C | 0.47 | 0.06 ~ 3.54 | 0.446 |  |  |  |  |
| I+T | 1.33 | 0.14 ~ 12.37 | 0.800 |  |  |  |  |
| I+C+T | 0.40 | 0.04 ~ 3.90 | 0.430 |  |  |  |  |
| **Number of metastases** |  |  |  |  |  |  |  |
| ≤1 | 1.00 (Reference) |  |  |  | 1.00 (Reference) |  |  |
| >1 | 0.38 | 0.15 ~ 0.99 | 0.047 |  | 0.42 | 0.16 ~ 1.11 | 0.080 |
| **HER2** |  |  |  |  |  |  |  |
| Negative | 1.00 (Reference) |  |  |  | 1.00 (Reference) |  |  |
| Positive | 0.36 | 0.16 ~ 0.84 | 0.018 |  | 0.39 | 0.17 ~ 0.92 | 0.031 |
| Unknown | 0.46 | 0.08 ~ 2.58 | 0.379 |  | 0.47 | 0.08 ~ 2.70 | 0.398 |
| **MMR** |  |  |  |  |  |  |  |
| Negative | 1.00 (Reference) |  |  |  |  |  |  |
| Positive | 3.05 | 0.48 ~ 19.33 | 0.236 |  |  |  |  |
| Unknown | 1.31 | 0.51 ~ 3.39 | 0.580 |  |  |  |  |
| **PD-L1** |  |  |  |  |  |  |  |
| Negative | 1.00 (Reference) |  |  |  |  |  |  |
| Positive | 1.44 | 0.57 ~ 3.66 | 0.441 |  |  |  |  |
| Unknown | 2.09 | 0.65 ~ 6.74 | 0.217 |  |  |  |  |

IrAEs, immune-related adverse events; AGC, advanced gastric cancer; Multi-irAEs, multi-organ irAEs; OR, odds ratio; CI, confidence interval; ECOG, Eastern Cooperative Oncology Group; TNM, tumor-node-metastasis; MMR, microsatellite mismatch repair HER2, human epidermal growth factor receptor-2; PD-L1, programmed cell death ligand 1.

**Table S4.** Response rate analysis of each organ-specific irAE vs. other patients in AGC patients.

| **Comparison** | PD | SD | PR | CR | DCR | *p-*value |  | ORR | *p-*value |
| --- | --- | --- | --- | --- | --- | --- | --- | --- | --- |
| **Others group vs. Thyroid irAE group** |  |  |  |  |  | 0.022 |  |  | 0.532 |
| Others | 76 | 132 | 41 | 0 | 69.5% |  |  | 16.5% |  |
| Thyroid irAEs | 5 | 26 | 7 | 1 | 87.2% |  |  | 20.5% |  |
| **Others group vs. Adrenal gland irAE group**  Others  Adrenal gland irAEs | 76  5 | 140  18 | 42  6 | 1  0 | 70.7%  82.8% | 0.169 |  | 16.6%  20.7% | 0.768 |
| **Others group vs. Heart irAE group** |  |  |  |  |  | 0.086 |  |  | 0.128 |
| Others | 73 | 150 | 48 | 1 | 73.2% |  |  | 18.0% |  |
| Heart irAE | 8 | 8 | 0 | 0 | 50.0% |  |  | 0.0% |  |
| **Others group vs. Lung irAE group** |  |  |  |  |  | 0.566 |  |  | 0.719 |
| Others | 79 | 151 | 45 | 1 | 71.4% |  |  | 16.7% |  |
| Lung irAE | 2 | 7 | 3 | 0 | 83.3% |  |  | 25.0% |  |
| **Others group vs. Skin irAE group** |  |  |  |  |  | 0.526 |  |  | 0.917 |
| Others | 73 | 138 | 43 | 0 | 71.3% |  |  | 16.9% |  |
| Skin irAE | 8 | 20 | 5 | 1 | 76.5% |  |  | 17.6% |  |

Other patients, including all the patients except the corresponding organ-specific irAE patients; IrAEs, immune-related adverse events; AGC, advanced gastric cancer; Vs, versus; SD, stable disease; PR, partial response; PD, progressive disease; CR, complete response; ORR, objective response rate; DCR, disease control rate.

**Table S5.** Median PFS and OS of each organ-specific group vs. other patients in AGC patients.

| **Covariate** | **Median PFS (95% CI)**  **(months)** | ***p-*value**  **(Log-rank)** | **Median OS (95% CI)**  **(months)** | ***p-*value**  **(Log-rank)** |
| --- | --- | --- | --- | --- |
| **Thyroid irAE** |  | 0.023 |  | 0.004 |
| Others | 5.77 (4.81 *~* 6.73) |  | 12.27 (10.75 *~* 13.78) |  |
| Thyroid | 11.37 (7.20 *~* 15.54) |  | 24.43 (9.61 *~* 39.25) |  |
| **Adrenal gland irAE**  Others  Adrenal | 5.83 (4.92 *~* 6.75)  10.23 (8.26 *~* 12.21) | 0.006 | 12.27 (11.05 ~ 13.48)  16.30 (14.70 ~ NA) | 0.003 |
| **Heart irAE**  Others  Heart | 6.60 (5.45 *~* 7.75)  3.53 (0.00 *~* 7.91) | 0.536 | 13.10 (11.56 ~ 14.64)  12.27 (10.00 ~ 14.54) | 0.916 |
| **Lung irAE**  Others  Lung | 6.43 (5.27 *~* 7.59)  6.27 (3.66 *~* 8.87) | 0.301 | 13.00 (11.55 *~* 14.45)  32.27 (0.00 *~* 82.12) | 0.180 |
| **Skin irAE**  Others  Skin | 5.83 (4.48 *~* 7.19)  7.40 (5.82 *~* 8.98) | 0.441 | 12.40 (10.99 *~* 13.81)  17.37 (14.36 *~* 20.37) | 0.016 |

Other patients, including all the patients except the corresponding organ-specific irAE patients; IrAEs, immune-related adverse events; AGC, advanced gastric cancer; Vs, versus; CI, confidence interval; OS, overall survival; PFS, progression free survival.

**Table S6.** Univariate analysis for the clinical characteristics associated with PFS in AGC patients (each organ-specific uni-irAE with a case number of more than 10 patients was included in clinical characteristics for analysis).

| **Covariate** | **Univariate Analysis** | |
| --- | --- | --- |
|  | **HR (95% CI)** | ***p-*value** |
| **Gender** |  |  |
| Female | 1.00 (Reference) |  |
| Male | 0.85 (0.63 ~ 1.14) | 0.278 |
| **Age** |  |  |
| 18-44 | 1.00 (Reference) |  |
| 45-65 | 0.53 (0.32 ~ 0.87) | 0.013 |
| ＞65 | 0.49 (0.30 ~ 0.81) | 0.006 |
| **ECOG** |  |  |
| ≤1 | 1.00 (Reference) |  |
| ＞1 | 1.71 (1.31 ~ 2.22) | <.001 |
| **TNM** |  |  |
| Ⅲ | 1.00 (Reference) |  |
| Ⅳ | 1.51 (1.11 ~ 2.04) | 0.008 |
| **Family history** |  |  |
| No | 1.00 (Reference) |  |
| Yes | 1.02 (0.75 ~ 1.39) | 0.875 |
| **Tumor site** |  |  |
| Non-cardia cancer | 1.00 (Reference) |  |
| Cardia cancer | 1.03 (0.79 ~ 1.33) | 0.845 |
| **Tumor differentiation** |  |  |
| Medium to high | 1.00 (Reference) |  |
| Low | 1.01 (0.77 ~ 1.32) | 0.970 |
| **Treatment line** |  |  |
| 1 | 1.00 (Reference) |  |
| >1 | 1.90 (1.46 ~ 2.48) | <.001 |
| **Treatment regimen** |  |  |
| I | 1.00 (Reference) |  |
| I+C | 1.56 (0.50 ~ 4.89) | 0.446 |
| I+T | 2.93 (0.89 ~ 9.67) | 0.077 |
| I+C+T | 2.39 (0.74 ~ 7.73) | 0.147 |
| **Number of metastases** |  |  |
| ≤1 | 1.00 (Reference) |  |
| >1 | 1.17 (0.89 ~ 1.53) | 0.267 |
| **HER2** |  |  |
| Negative | 1.00 (Reference) |  |
| Positive | 0.76 (0.58 ~ 0.99) | 0.046 |
| Unknown | 0.62 (0.35 ~ 1.09) | 0.099 |
| **MMR** |  |  |
| Negative | 1.00 (Reference) |  |

**Table S6. continued.**

| Positive | 0.35 (0.13 ~ 0.95) | 0.038 |  |
| --- | --- | --- | --- |
| Unknown | 1.00 (0.72 ~ 1.40) | 0.989 |  |
| **PD-L1** |  |  |  |
| Negative | 1.00 (Reference) |  |  |
| Positive | 0.70 (0.52 ~ 0.95) | 0.021 |  |
| Unknown | 0.89 (0.59 ~ 1.35) | 0.578 |  |
| **Thyroid irAE** |  |  |  |
| Others | 1.00 (Reference) |  |  |
| Thyroid | 0.65 (0.44 ~ 0.95) | 0.025 |  |
| **Adrenal gland** **irAE** |  |  |  |
| Others | 1.00 (Reference) |  |  |
| Adrenal gland | 0.50 (0.31 ~ 0.83) | 0.007 |  |
| **Heart irAE** |  |  |  |
| Others | 1.00 (Reference) |  |  |
| Heart | 1.19 (0.68 ~ 2.09) | 0.537 |  |
| **Lung irAE** |  |  |  |
| Others | 1.00 (Reference) |  |  |
| Lung | 0.70 (0.36 ~ 1.38) | 0.304 |  |
| **Skin irAE** |  |  |  |
| Others | 1.00 (Reference) |  |  |
| Skin | 0.85 (0.57 ~ 1.28) | 0.442 |  |

IrAEs, immune-related adverse events; Uni-irAE, single-organ irAE; AGC, advanced gastric cancer; HR, hazard ratio; CI, confidence interval; PFS, progression free survival; ECOG, Eastern Cooperative Oncology Group; TNM, tumor-node-metastasis; MMR, microsatellite mismatch repair; HER2, human epidermal growth factor receptor-2; PD-L1, programmed cell death ligand 1.

**Table S7.** Univariate analysis for the clinical characteristics associated with OS in AGC patients (each organ-specific uni-irAE with a case number of more than 10 patients was included in clinical characteristics for analysis).

| **Covariate** | **Univariate Analysis** | |
| --- | --- | --- |
|  | **HR (95% CI)** | ***p*-value** |
| **Gender** |  |  |
| Female | 1.00 (Reference) |  |
| Male | 0.98 (0.71 ~ 1.35) | 0.885 |
| **Age** |  |  |
| 18-44 | 1.00 (Reference) |  |
| 45-65 | 0.47 (0.28 ~ 0.79) | 0.004 |
| ＞65 | 0.47 (0.28 ~ 0.79) | 0.005 |
| **ECOG** |  |  |
| ≤1 | 1.00 (Reference) |  |
| ＞1 | 1.66 (1.25 ~ 2.21) | <.001 |
| **TNM** |  |  |
| Ⅲ | 1.00 (Reference) |  |
| Ⅳ | 1.70 (1.19 ~ 2.42) | 0.003 |
| **Family history** |  |  |
| No | 1.00 (Reference) |  |
| Yes | 1.04 (0.74 ~ 1.45) | 0.842 |
| **Tumor site** |  |  |
| Non-cardia cancer | 1.00 (Reference) |  |
| Cardia cancer | 0.92 (0.69 ~ 1.22) | 0.562 |
| **Tumor differentiation** |  |  |
| Medium to high | 1.00 (Reference) |  |
| Low | 1.03 (0.76 ~ 1.39) | 0.844 |
| **Treatment line** |  |  |
| 1 | 1.00 (Reference) |  |
| >1 | 1.90 (1.42 ~ 2.53) | <.001 |
| **Treatment regimen** |  |  |
| I | 1.00 (Reference) |  |
| I+C | 0.98 (0.31 ~ 3.08) | 0.972 |
| I+T | 1.49 (0.45 ~ 4.95) | 0.516 |
| I+C+T | 1.45 (0.45 ~ 4.75) | 0.535 |
| **Number of metastases** |  |  |
| ≤1 | 1.00 (Reference) |  |
| >1 | 1.13 (0.84 ~ 1.52) | 0.426 |
| **HER2** |  |  |
| Negative | 1.00 (Reference) |  |
| Positive | 0.79 (0.59 ~ 1.06) | 0.117 |
| Unknown | 0.72 (0.39 ~ 1.32) | 0.284 |
| **MMR** |  |  |
| Negative | 1.00 (Reference) |  |

**Table S7. continued.**

| Positive | 0.52 (0.19 ~ 1.41) | 0.199 |
| --- | --- | --- |
| Unknown | 0.91 (0.63 ~ 1.32) | 0.631 |
| **PD-L1** |  |  |
| Negative | 1.00 (Reference) |  |
| Positive | 0.84 (0.60 ~ 1.17) | 0.301 |
| Unknown | 0.84 (0.53 ~ 1.33) | 0.456 |
| **Thyroid irAE** |  |  |
| Others | 1.00 (Reference) |  |
| Thyroid | 0.52 (0.33 ~ 0.82) | 0.005 |
| **Adrenal gland** **irAE** |  |  |
| Others | 1.00 (Reference) |  |
| Adrenal gland | 0.42 (0.24 ~ 0.76) | 0.004 |
| **Heart irAE** |  |  |
| Others | 1.00 (Reference) |  |
| Heart | 0.97 (0.51 ~ 1.83) | 0.916 |
| **Lung irAE** |  |  |
| Others | 1.00 (Reference) |  |
| Lung | 0.58 (0.25 ~ 1.30) | 0.186 |
| **Skin irAE** |  |  |
| Others | 1.00 (Reference) |  |
| Skin | 0.54 (0.32 ~ 0.90) | 0.018 |

IrAEs, immune-related adverse events; Uni-irAE, single-organ irAE; AGC, advanced gastric cancer; OS, overall survival; HR, hazard ratio; CI, confidence interval; PFS, progression free survival; ECOG, Eastern Cooperative Oncology Group; TNM, tumor-node-metastasis; MMR, microsatellite mismatch repair; HER2, human epidermal growth factor receptor-2; PD-L1, programmed cell death ligand 1.

**Table S8.** Multivariate Cox proportional hazard model analysis for the association between PFS and thyroid irAE in AGC patients.

| **Covariate** | **Multivariate Analysis** | |
| --- | --- | --- |
|  | **HR (95% CI)** | ***p-*value** |
| **Age** |  |  |
| 18-44 | 1.00 (Reference) |  |
| 45-65 | 0.42 (0.25 ~ 0.71) | 0.001 |
| ＞65 | 0.45 (0.27 ~ 0.76) | 0.003 |
| **ECOG** |  |  |
| ≤1 | 1.00 (Reference) |  |
| ＞1 | 1.58 (1.20 ~ 2.07) | 0.001 |
| **TNM** |  |  |
| Ⅲ | 1.00 (Reference) |  |
| Ⅳ | 1.28 (0.93 ~ 1.76) | 0.123 |
| **Treatment line** |  |  |
| 1 | 1.00 (Reference) |  |
| >1 | 2.00 (1.51 ~ 2.66) | <.001 |
| **HER2** |  |  |
| Negative | 1.00 (Reference) |  |
| Positive | 0.81 (0.62 ~ 1.07) | 0.135 |
| Unknown | 0.72 (0.37 ~ 1.39) | 0.329 |
| **MMR** |  |  |
| Negative | 1.00 (Reference) |  |
| Positive | 0.48 (0.17 ~ 1.31) | 0.152 |
| Unknown | 1.02 (0.68 ~ 1.53) | 0.922 |
| **PD-L1** |  |  |
| Negative | 1.00 (Reference) |  |
| Positive | 0.75 (0.54 ~ 1.04) | 0.081 |
| Unknown | 0.85 (0.52 ~ 1.40) | 0.531 |
| **Thyroid irAE** |  |  |
| Others | 1.00 (Reference) |  |
| Thyroid | 0.65 (0.44 ~ 0.96) | 0.031 |

IrAEs, immune-related adverse events; AGC, advanced gastric cancer; HR, hazard ratio; CI, confidence interval; PFS, progression free survival; ECOG, Eastern Cooperative Oncology Group; TNM, tumor-node-metastasis; MMR, microsatellite mismatch repair; HER2, human epidermal growth factor receptor-2; PD-L1, programmed cell death ligand 1.

**Table S9.** Multivariate Cox proportional hazard model analysis for the association between PFS and adrenal gland irAE in AGC patients.

| **Covariate** | **Multivariate Analysis** | |
| --- | --- | --- |
|  | **HR (95% CI)** | ***p-*value** |
| **Age** |  |  |
| 18-44 | 1.00 (Reference) |  |
| 45-65 | 0.39 (0.23 ~ 0.66) | <.001 |
| ＞65 | 0.42 (0.25 ~ 0.71) | 0.001 |
| **ECOG** |  |  |
| ≤1 | 1.00 (Reference) |  |
| ＞1 | 1.70 (1.29 ~ 2.25) | <.001 |
| **TNM** |  |  |
| Ⅲ | 1.00 (Reference) |  |
| Ⅳ | 1.26 (0.92 ~ 1.72) | 0.156 |
| **Treatment line** |  |  |
| 1 | 1.00 (Reference) |  |
| >1 | 1.79 (1.35 ~ 2.38) | <.001 |
| **HER2** |  |  |
| Negative | 1.00 (Reference) |  |
| Positive | 0.81 (0.61 ~ 1.06) | 0.126 |
| Unknown | 0.65 (0.33 ~ 1.28) | 0.212 |
| **MMR** |  |  |
| Negative | 1.00 (Reference) |  |
| Positive | 0.47 (0.17 ~ 1.30) | 0.145 |
| Unknown | 1.06 (0.71 ~ 1.58) | 0.783 |
| **PD-L1** |  |  |
| Negative | 1.00 (Reference) |  |
| Positive | 0.74 (0.54 ~ 1.02) | 0.069 |
| Unknown | 0.89 (0.54 ~ 1.46) | 0.635 |
| **Adrenal gland** **irAE** |  |  |
| Others | 1.00 (Reference) |  |
| Adrenal gland | 0.50 (0.30 ~ 0.83) | 0.008 |

IrAEs, immune-related adverse events; AGC, advanced gastric cancer; HR, hazard ratio; CI, confidence interval; PFS, progression free survival; ECOG, Eastern Cooperative Oncology Group; TNM, tumor-node-metastasis; MMR, microsatellite mismatch repair; HER2, human epidermal growth factor receptor-2; PD-L1, programmed cell death ligand 1.

**Table S10.** Multivariate Cox proportional hazard model analysis for the association between OS and thyroid irAE in AGC patients.

| **Covariate** | **Multivariate Analysis** | |
| --- | --- | --- |
|  | **HR (95% CI)** | ***p-*value** |
| **Age** |  |  |
| 18-44 | 1.00 (Reference) |  |
| 45-65 | 0.43 (0.25 ~ 0.73) | 0.002 |
| ＞65 | 0.48 (0.28 ~ 0.83) | 0.009 |
| **ECOG** |  |  |
| ≤1 | 1.00 (Reference) |  |
| ＞1 | 1.50 (1.12 ~ 2.01) | 0.006 |
| **TNM** |  |  |
| Ⅲ | 1.00 (Reference) |  |
| Ⅳ | 1.47 (1.02 ~ 2.13) | 0.039 |
| **Treatment line** |  |  |
| 1 | 1.00 (Reference) |  |
| >1 | 1.92 (1.43 ~ 2.58) | <.001 |
| **Thyroid irAE** |  |  |
| Others | 1.00 (Reference) |  |
| Thyroid | 0.53 (0.34 ~ 0.84) | 0.007 |

IrAEs, immune-related adverse events; AGC, advanced gastric cancer; OS, overall survival; HR, hazard ratio; CI, confidence interval; ECOG, Eastern Cooperative Oncology Group; TNM, tumor-node-metastasis.

**Table S11.** Multivariate Cox proportional hazard model analysis for the association between OS and adrenal gland irAE in AGC patients.

| **Covariate** | **Multivariate Analysis** | |
| --- | --- | --- |
|  | **HR (95% CI)** | ***p-*value** |
| **Age** |  |  |
| 18-44 | 1.00 (Reference) |  |
| 45-65 | 0.38 (0.22 ~ 0.65) | <.001 |
| ＞65 | 0.44 (0.25 ~ 0.75) | 0.003 |
| **ECOG** |  |  |
| ≤1 | 1.00 (Reference) |  |
| ＞1 | 1.62 (1.21 ~ 2.17) | 0.001 |
| **TNM** |  |  |
| Ⅲ | 1.00 (Reference) |  |
| Ⅳ | 1.44 (1.01 ~ 2.08) | 0.049 |
| **Treatment line** |  |  |
| 1 | 1.00 (Reference) |  |
| >1 | 1.71 (1.28 ~ 2.30) | <.001 |
| **Adrenal gland** **irAE** |  |  |
| Others | 1.00 (Reference) |  |
| Adrenal gland | 0.47 (0.26 ~ 0.85) | 0.012 |

IrAEs, immune-related adverse events; AGC, advanced gastric cancer; OS, overall survival; HR, hazard ratio; CI, confidence interval; ECOG, Eastern Cooperative Oncology Group; TNM, tumor-node-metastasis.

**Table S12.** Multivariate Cox proportional hazard model analysis for the association between OS and skin irAE in AGC patients.

| **Covariate** | **Multivariate Analysis** | |
| --- | --- | --- |
|  | **HR (95% CI)** | ***p-*value** |
| **Age** |  |  |
| 18-44 | 1.00 (Reference) |  |
| 45-65 | 0.39 (0.23 ~ 0.66) | <.001 |
| ＞65 | 0.45 (0.26 ~ 0.77) | 0.003 |
| **ECOG** |  |  |
| ≤1 | 1.00 (Reference) |  |
| ＞1 | 1.54 (1.15 ~ 2.07) | 0.004 |
| **TNM** |  |  |
| Ⅲ | 1.00 (Reference) |  |
| Ⅳ | 1.51 (1.05 ~ 2.18) | 0.028 |
| **Treatment line** |  |  |
| 1 | 1.00 (Reference) |  |
| >1 | 1.76 (1.31 ~ 2.37) | <.001 |
| **Skin irAE** |  |  |
| Others | 1.00 (Reference) |  |
| Skin | 0.58 (0.35 ~ 0.97) | 0.039 |

IrAEs, immune-related adverse events; AGC, advanced gastric cancer; OS, overall survival; HR, hazard ratio; CI, confidence interval; ECOG, Eastern Cooperative Oncology Group; TNM, tumor-node-metastasis.

**Figure S1. A.-J.** The Kaplan–Meier curve of progression-free survival (PFS) and overall survival (OS) of each specific irAE with a case number of more than 10 patients in AGC patients. A: PFS of thyroid irAE patients. B: OS of thyroid irAE patients. C: PFS of adrenal gland irAE patients. D: OS of adrenal gland irAE patients. E: PFS of heart irAE patients. F: OS of heart irAE patients. G: PFS of skin irAE patients. H: OS of skin irAE patients. I: PFS of lung irAE patients. J: OS of lung irAE patients.

**
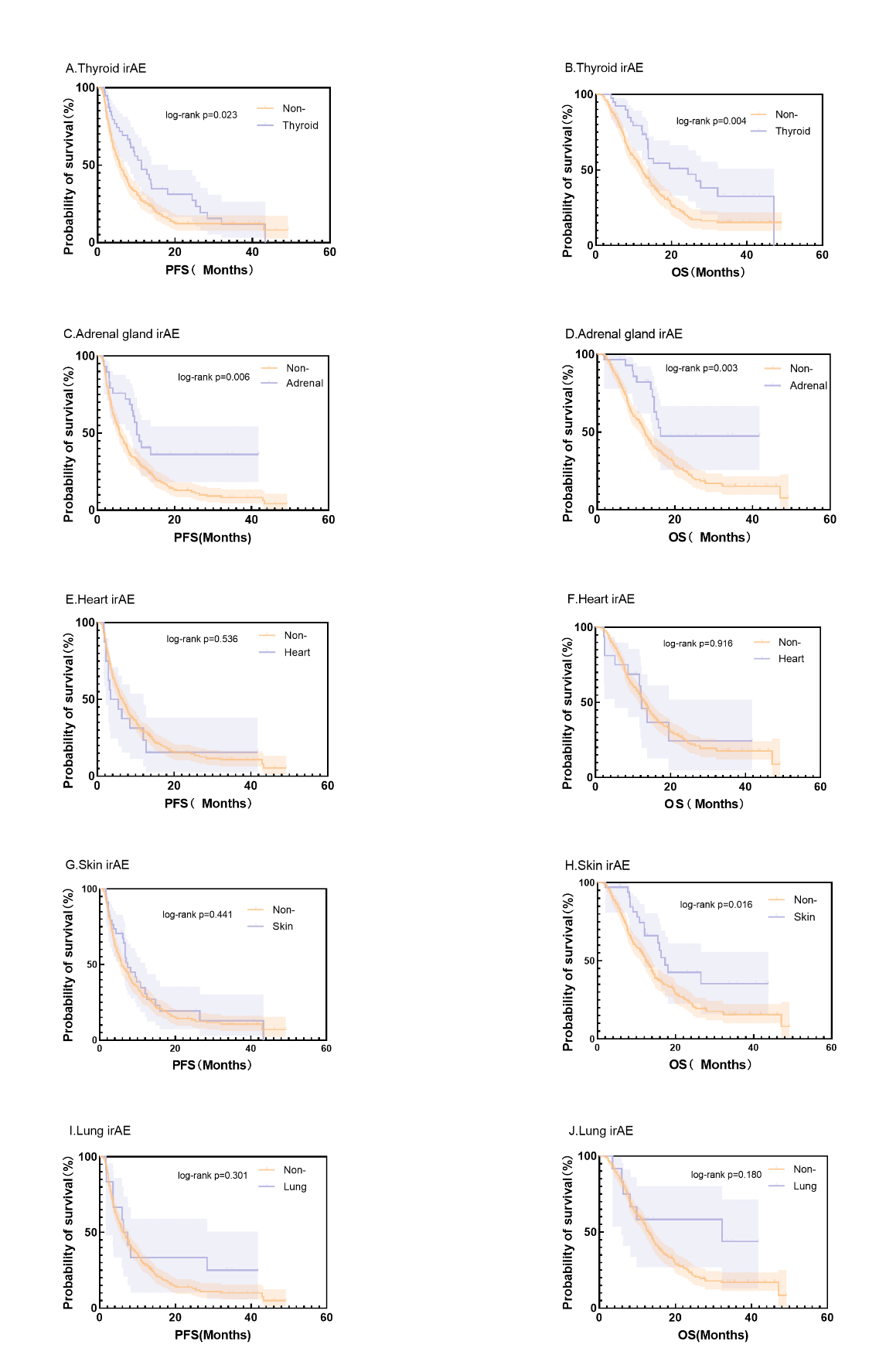
**
